# Acute Renal Failure and Severe Hypertension from a Page Kidney Post-Transplant Biopsy

**DOI:** 10.1100/tsw.2010.150

**Published:** 2010-08-03

**Authors:** Maria Aurora Posadas, Vincent Yang, Bing Ho, Muhammad Omer, Daniel Batlle

**Affiliations:** Division of Nephrology and Hypertension, Department of Medicine, Feinberg School of Medicine, Northwestern University, Chicago, USA

**Keywords:** Page kidney, transplant biopsy, renin-angiotensin-aldosterone

## Abstract

Page kidney refers to a clinical picture characterized by acute onset of hypertension due to external compression of the kidneys from hematoma, tumor, lymphocele, or urinoma. Hypertension is believed to result from renin-angiotensin-aldosterone activation triggered by renal hypoperfusion and microvascular ischemia. Renal failure, in addition to hypertension, may occur in the setting of a single functional kidney or a diseased contralateral kidney. We report a case of a patient who had a transplant kidney biopsy complicated by a subcapsular perinephric hematoma. The patient presented with an acute increase in blood pressure and a rapid rise in serum creatinine following a transplant kidney routine biopsy. He underwent emergent evacuation of the perinephric hematoma, with consequent decrease of his blood pressure and return of serum creatinine back to his baseline level. Early recognition and rapid intervention are needed in order to correct hypertension and reverse acute renal failure in Page kidney occurring in renal transplant recipients.

## CASE REPORT

A 55-year-old male with a history of hypertension, diabetes, and cryptogenic cirrhosis complicated by hepatorenal syndrome developed renal failure and was started on hemodialysis in February 2009. He underwent liver and kidney transplantation in October 2009. Post-transplantation, hepatic and renal function had normalized and his plasma creatinine had decreased to 0.7–0.9 mg/dL. In January 2010, the patient underwent protocol allograft kidney biopsy, after which he developed abdominal pain at the biopsy site. His blood pressure, which is usually 140/70, increased rapidly to 200/100 mmHg. A renal panel showed that his serum creatinine had increased to 3.5 mg/dL. A transplant kidney ultrasound revealed a large subcapsular perinephric hematoma and a concurrent Doppler study revealed elevated resistive indices (0.92–0.98 ratio) in the three poles of the allograft kidney due to the compressive effect of the large hematoma (Fig. 1).

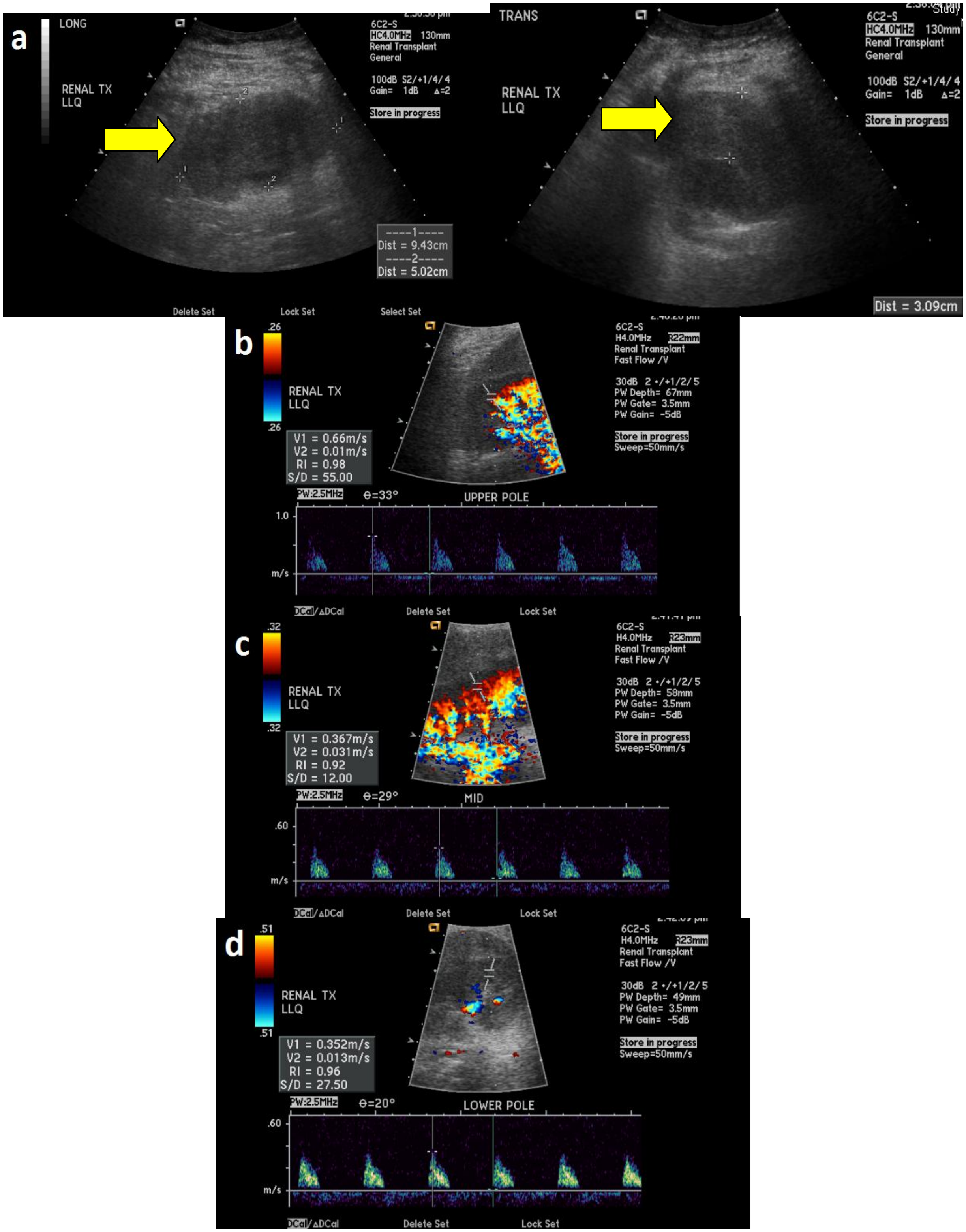

**FIGURE 1.** Transplant kidney ultrasound showing a large subcapsular perinephric concentric hematoma (a: marked by crosses and yellow arrows). Doppler studies showed increased resistive indices on the upper (b), middle (c), and lower (d) poles of the kidney.

The patient underwent an emergency exploration of the transplant kidney and evacuation of the hematoma. After surgical intervention, his blood pressure decreased to baseline, and his serum creatinine fell rapidly and returned to baseline (Fig. 2).

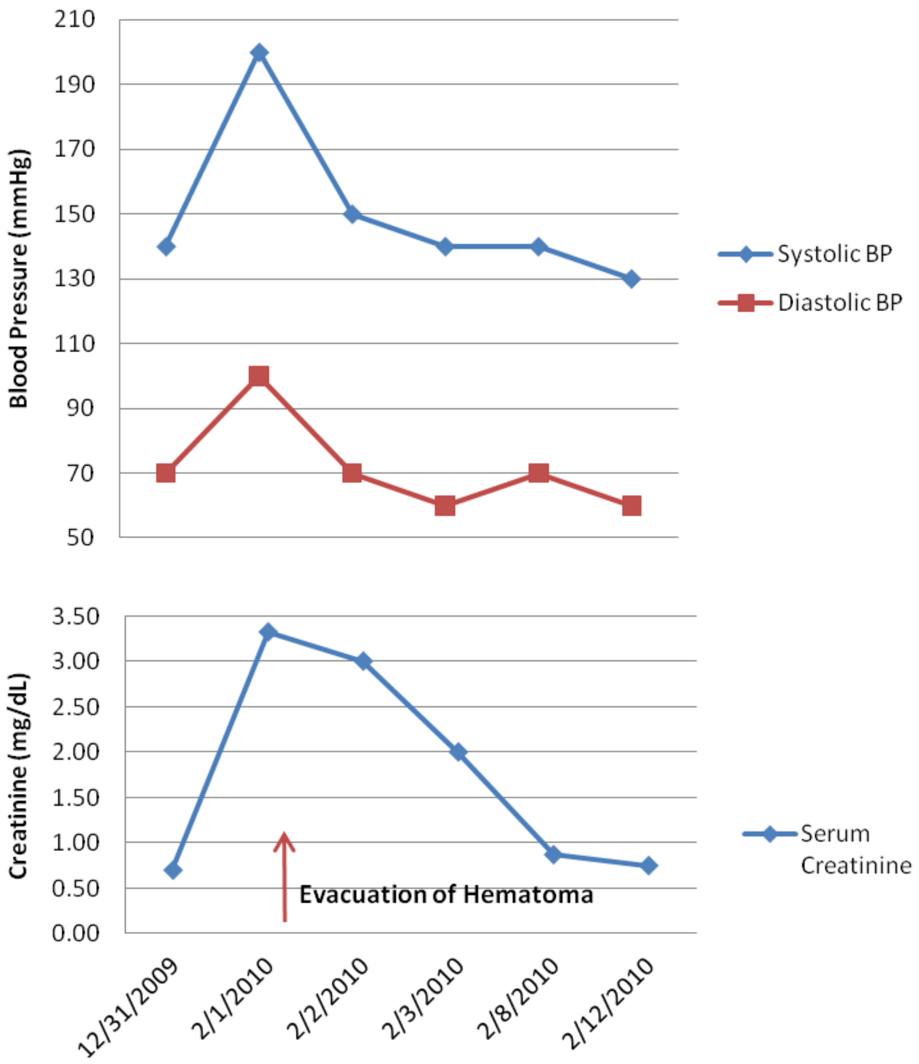

**FIGURE 2.** Blood pressure and creatinine before and after the kidney biopsy and subsequent evacuation of the kidney hematoma on an emergency basis on the evening of 2/1/2010.

## DISCUSSION

Page kidney was first described by Irvine Page in 1939, when he wrapped animal kidneys with cellophane and observed the development of acute hypertension[1]. The first clinical case of Page kidney was described in 1955 by Engel and Page in an American football player who had blunt trauma resulting in renal hematoma and subsequent hypertension[2]. Since then, Page kidney has been shown to result from several precipitating events, including trauma from sports and motor vehicle accidents, lithotripsy, kidney biopsy, tumors, lymphoceles, and urinomas[3]. Page kidney in a kidney transplant has been recognized more recently[4,5,6]. Hypertension has been ascribed to result from renal hypoperfusion and microvascular ischemia from external compression of the kidney, and subsequent activation of the reninangiotensin-aldosterone system. Recent experimental studies have shown that interstitial inflammation may be the primary cause[7]. Regardless of the pathogenesis, our case illustrates very well the rapid reversibility of the hypertension with relief of the hematoma. Moreover, it shows that in the setting of a single functioning kidney or a diseased contralateral kidney, renal failure can occur and also be readily reversible. As shown in Fig. 2, evacuation of the hematoma caused by the kidney biopsy resulted not only in a resolution of the hypertensive crisis, but also a decline in serum creatinine.

In summary, acute kidney injury due to Page kidney developing in a solitary kidney is potentially reversible if recognized early. Several treatment modalities have been employed to treat Page kidney associated with large renal hematomas, including evacuation of hematoma, decapsulation, and nephrectomy[3,5,6]. Success with medical management of hypertension with ACEI[8] and diuretics[9] has also been reported. In the presence of renal failure in a solitary Page kidney, however, aggressive and prompt intervention is warranted to treat acute kidney injury, as this case illustrates.
